# World-Class Male Sprinters and High Hurdlers Have Similar Start and Initial Acceleration Techniques

**DOI:** 10.3389/fspor.2019.00023

**Published:** 2019-09-18

**Authors:** Ian N. Bezodis, Adam Brazil, Hans C. von Lieres und Wilkau, Matthew A. Wood, Giorgios P. Paradisis, Brian Hanley, Catherine B. Tucker, Lysander Pollitt, Stéphane Merlino, Pierre-Jean Vazel, Josh Walker, Athanassios Bissas

**Affiliations:** ^1^Cardiff School of Sport and Health Sciences, Cardiff Metropolitan University, Cardiff, United Kingdom; ^2^Department for Health, University of Bath, Bath, United Kingdom; ^3^Athletics Sector, School of Physical Education and Sport Science, National and Kapodistrian University of Athens, Athens, Greece; ^4^Carnegie School of Sport, Leeds Beckett University, Leeds, United Kingdom; ^5^Development Department, International Association of Athletics Federations, Monaco City, Monaco; ^6^Athlétisme Metz Métropole, Metz, France

**Keywords:** acceleration, biomechanics, coordination, hurdles, kinematics, sprint start, track and field, world championships

## Abstract

The effect of the inclusion of a high hurdle 13.72 m after the start line on elite sprint start and initial acceleration technique has yet to be investigated or understood. This highly novel study addresses that lack of information in an exceptional manner, through detailed biomechanical analysis of the world's best sprint and hurdle athletes, with data collected *in situ* at the 2018 IAAF World Indoor Championships, held in Birmingham, UK. High speed videos (150 Hz) were compared for eight sprinters and seven hurdlers for the start and initial acceleration phase of the finals of the men's 60 m and 60 m hurdles. Temporal and kinematic data were supplemented by vector coding analysis to investigate mechanisms by which these world-class athletes translate their centres of mass (CM) up to the fourth touchdown post-block exit. The sprinters and hurdlers coordinated their lower limb and trunk movement in a similar manner throughout the start and initial acceleration phases, which contributes new conceptual understanding of the mechanisms that underpin start and initial acceleration performance. Differences between groups were initiated from block set-up, with the hurdlers utilising a larger block spacing, but with the front block nearer to the start line than sprinters. Even after accounting for stature, the biggest differences in the raising of the CM occurred during the block phase, with hurdlers greater than sprinters (difference in vertical CM displacement scaled to stature = −0.037, very large effect size). Subsequent flight phases showed the biggest differences in the translation of the CM, in part due to longer flight times in the hurdlers, whilst the techniques of the two groups generally converged during the ground contact phases of initial acceleration. In highlighting that similar techniques are used by world-class sprinters and hurdlers, despite differing task constraints, this study has provided invaluable insights for scientists, coaches, and athletes, that will inform further developments in understanding and practice across both sprints and hurdles.

## Introduction

Although it is well-established that effective maximal sprint acceleration is dependent on primarily horizontal external kinetics (Morin et al., 2011; Rabita et al., 2015) and matching segmental kinematics (Kugler and Janshen, 2010; Nagahara et al., 2014; von Lieres und Wilkau et al., 2018), research has not examined what effect the inclusion of a high hurdle has on acceleration performance and centre of mass (CM) projection. To the authors' knowledge the only published works investigating the initial acceleration phase in sprint hurdlers have been confined to a simple spatio-temporal analysis (Rash et al., 1990) and a single athlete case study covering kinematics, external kinetics, and electromyography (Coh et al., 2007) in female athletes, for whom the hurdles are 0.838 m high. For male high hurdlers, the placement of a 1.067 m high hurdle 13.72 m from the start line introduces an additional task constraint. This is yet to be investigated in terms of its effect on performance in the start and initial acceleration phase, which are critical to optimal overall performance. The initial acceleration phase has previously been defined as lasting four to six steps after block exit (Nagahara et al., 2014; von Lieres und Wilkau et al., 2018).

A key characteristic of a hurdler's approach to the first hurdle is the number of steps taken, which in recent years has shifted to a seven-step pattern among the world's elite. However, the coaching literature has suggested both seven (Freeman, 2015) and eight step approaches (Mann and Murphy, 2018) as closer to initial acceleration mechanics in sprinters. The absence of an examination of initial acceleration mechanics in high hurdles and a detailed comparison of sprint and hurdle start and initial acceleration has important implications for both coach and athlete development as well as the understanding of optimal performance.

The word “elite” is overused in sports science literature, and the number of published studies on the biomechanics of the world's best able-bodied sprinters is small. Out-of-competition data have been presented from the block start (Bezodis et al., 2015; Willwacher et al., 2016), initial acceleration phase (Wild et al., 2018), composite 40 m maximal acceleration (Rabita et al., 2015), maximum velocity phase (Bezodis et al., 2008, 2018), and a full 100 m sprint (Morin et al., 2012) of groups of athletes that generally contained one sub-10 s sprinter. There are also examples of analyses of elite 100 m races based primarily on distance-time data, either taken from broadcast television footage or data from previous IAAF biomechanics projects (e.g., Salo et al., 2011; Taylor and Beneke, 2012; Slawinski et al., 2017). However, to the authors' knowledge, more detailed kinematic analyses of elite athletes in competition are confined to the block and initial acceleration phase from a Diamond League 100 m final (Ciacci et al., 2017) and the home straight of the 1984 Olympic 200 m final (Mann and Herman, 1985b). Lately, the IAAF has published detailed biomechanical reports from both outdoor and indoor World Championships (Bissas et al., 2018; Pollitt et al., 2018; Walker et al., 2019a,b). However, the lack of peer-reviewed analysis of “world-class sprinters (e.g., international finalists)” has very recently been highlighted in a comprehensive review of the biomechanics of the sprint start as a major gap in the research literature (Bezodis et al., 2019b).

The inherent nature of biomechanical data collection with elite athletes in competition necessitates an approach that minimises interference with the athletes. This usually means that studies conducted in these settings have to focus on kinematic analyses (e.g., Mann and Herman, 1985a,b; Ciacci et al., 2017). Studies of sub-elite athletes have demonstrated that segmental orientations closely reflect the external force characteristics that are important for effective maximal sprint acceleration (Kugler and Janshen, 2010; Nagahara et al., 2014; von Lieres und Wilkau et al., 2018). Therefore, kinematic analyses can play an important role in improving the understanding of the mechanisms that underpin effective sprint start and acceleration performance. The role of the trunk segment during the block phase (Slawinski et al., 2012), and of the shank and trunk segments during the initial acceleration phase (von Lieres und Wilkau et al., 2018) have been shown to be important for efficient performance. At a joint kinetic level in the initial acceleration phase, the ankle plantarflexors and hip extensors are important energy generators (Charalambous et al., 2012; Bezodis et al., 2014; Brazil et al., 2017), whilst the magnitude of knee extensor energy generation is thought to relate to sprint performance (Debaere et al., 2013; Bezodis et al., 2014; Brazil et al., 2018).

Studies of the block and initial acceleration phases of sprinters have shown mechanisms by which they project their CM to address the task. Debaere et al. (2013) reported horizontal and vertical block exit velocities of 3.10 and 0.84 m/s, respectively. Whereas the horizontal velocity increased in a relatively consistent linear manner throughout the block phase, the vertical velocity increased rapidly through the initial double-leg push phase, peaking at 0.74 m/s, and then only showing a small further increase up to block exit. This suggests that there is an equal focus on both forward and upward projection of the CM during the double-leg push phase, but that this switches to a primary focus on the forward projection of the CM during the single-leg push phase. Characteristics of external force data gathered during the block phase support this suggestion (Willwacher et al., 2016; Bezodis et al., 2019a), and it has previously been shown that rear-leg force production in the blocks is a key discriminant of sprint performance in athletes ranging from national level to world-class (Fortier et al., 2005; Willwacher et al., 2016; Brazil et al., 2018).

The assessment of coordination offers an advancement beyond single-joint kinematic analysis to understand sports technique, offering insight into the interaction between components of the biological system that are functionally linked to satisfy the demands of a given task (Bernstein, 1967; Turvey, 1990). The theoretical model of constraints on action (Newell, 1986) describes how individuals adopt movement coordination patterns via self-organisation within the context of organismic, environmental and task related constraints imposed on the biological system. These coordination patterns are commonly assessed through investigating the relative motion between joints or segments of the same limb, providing a measure of intra-limb coordination (Sparrow et al., 1987) that can improve understanding of how gross movement is organised, and for gait, therefore, how the translation of the CM is controlled. Intra-limb coordination analyses have been applied to constant velocity locomotive task such as walking (Chang et al., 2008), running (Hamill et al., 1999; Floría et al., 2019), and maximal velocity sprinting (Gittoes and Wilson, 2010). Vector coding methods (Sparrow et al., 1987; Chang et al., 2008; Needham et al., 2014) output a coupling angle, which can be easily related back to angular motion, providing an intuitive applied method for assessing movement coordination. To the authors' knowledge, coordination analyses have not yet been applied to the block and initial acceleration phases of a maximal sprint in an elite population. Given the additional task constraint of the high hurdle, inter-segment coordination analyses will afford greater insight into technique differences between elite sprint and hurdle athletes.

The purpose of this study was therefore to address the gaps in the research literature in an exceptional way, based on detailed biomechanical data collected from the finals of the 2018 IAAF World Indoor Championships. This is the first time in the biomechanics research literature that such data have been captured live, enabling a novel approach to examining an important aspect of sprint acceleration performance. The first aim of the study was to quantify and explain the start and initial acceleration technique of the world's best male sprinters and hurdlers *in situ* in an elite competition environment. Secondly, based on a comparison of the sprinters and hurdlers, the aim was to elucidate the mechanisms by which the athletes translate their CM during the start and initial acceleration phases, given the different task constraints placed on the athletes by the two events. Findings from this study will contribute new conceptual understanding of the mechanisms that underpin start and initial acceleration performance, for scientists, coaches and athletes.

## Materials and Methods

### Participants

Data were collected as part of the Birmingham 2018 IAAF World Indoor Championships Biomechanics Research Project. The use of the data for this study was approved by the IAAF, who own and control the data, and locally through institutional research ethics procedures. The study was approved by the Leeds Beckett University Ethics sub-committee (School local approval by Research Ethics Coordinator). The patients/participants provided their written informed consent to participate in this study. The fifteen finalists of the men's 60 m and 60 m hurdles races (eight sprinters and seven hurdlers, because of a false start) were analysed in their respective races, on the evenings of 3rd and 4th March 2018 at Arena Birmingham, UK. All hurdlers took a seven-step approach to the first hurdle.

### Data Collection

**Figure 1:**
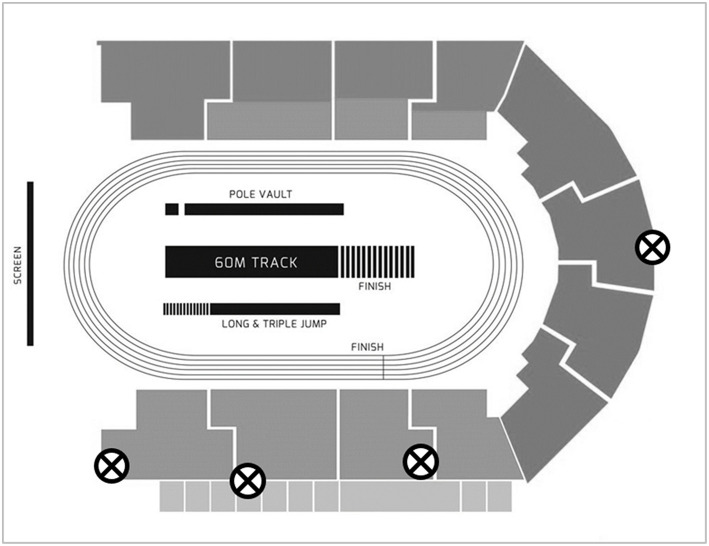
Camera positions for data capture. The four cameras are each marked with an ⊗.

Four Sony PXW-FS7 cameras operating at 150 Hz (shutter speed: 1/1250 s; ISO: 2000–4000; FHD: 1920 × 1080 pixels) were used to capture motion of athletes during block and initial acceleration phases (see Figure 1). A calibration procedure was conducted before and after each race. A rigid cuboid calibration frame measuring 3.044 × 3.044 × 3.044 m and comprising 24 control points was used. It was sequentially positioned multiple times over discrete predefined areas along and across the track to ensure an accurate definition of a volume covering the starting blocks and initial acceleration phase of the race, from 1 m behind the start line to 5 m beyond the start line. This approach produced a large number of non-coplanar control points per individual calibrated volume and facilitated the construction of a local coordinate system in each neighbouring pair of lanes, that was then combined into a global coordinate system, originating 1 m behind the left edge of lane 1.

### Data Processing

The video files were imported into SIMI Motion (SIMI Motion version 9.2.2, Simi Reality Motion Systems GmbH, Germany) and were manually digitised by a single experienced operator to obtain kinematic data. An event synchronisation technique (synchronisation of four critical instants) was applied through SIMI Motion to synchronise the two-dimensional coordinates from each camera involved in the recording. The digitising was carried out in two parts: a whole body analysis of specific discrete key events, and a continuous analysis of the trunk and rear leg throughout the start and initial acceleration phase. Firstly, a 17 point whole body model was digitised at the following key events; first visible movement from the set position (FM), rear foot block exit (RFBE), front foot block exit (FFBE), and touchdown and take-off events up to the touchdown of ground contact four (GC1_TD_, GC1_TO_ etc…see Table 1). From block exit onwards, these events were defined as the last frame where the foot was visibly on the block or track, and the subsequent first frame where the foot was visibly on the track, respectively. The 17 digitised points were the centre of the head, and bilaterally shoulder, elbow, wrist, metacarpo-phalangeal, hip, knee, ankle, and metatarso-phalangeal (MTP) joint centres in accordance with de Leva (1996).

**Table 1:** Abbreviations used in the study.

**Abbreviation**	**Meaning**
CM	Centre of mass
FM	First movement
RFBE	Rear foot block exit
FFBE	Front foot block exit
GC1, GC2, GC3	Ground contact one, two and three
GC1_TD_, etc…	Ground contact one touchdown, etc…
GC1_TO_, etc…	Ground contact one take-off, etc…
MTP	Metatarso-phalangeal
SD	Standard deviation
CA	Coupling angle
CA_DIF_	Coupling angle difference

Secondly, the shoulder, hip, knee, ankle, and MTP joints on the side of the rear leg in the blocks were digitised continuously in every frame from the onset of movement in the blocks (FM) to the third take-off after block exit (GC3_TO_). Each video file was digitised frame by frame and upon completion, adjustments were made as necessary using the points over frame method (Bahamonde and Stevens, 2006). The Direct Linear Transformation algorithm (Abdel-Aziz and Karara, 1971) was used to reconstruct the three-dimensional coordinates from individual camera's x and y image coordinates. For all subsequent analysis, three-dimensional coordinates were projected onto a two-dimensional sagittal plane using only antero-posterior and vertical coordinates. Reliability of the digitising process was estimated by repeating the process for specific variables for eight randomly selected athletes with an intervening period of 48 h. The results showed minimal total errors (CM vertical coordinate in the set position: RMSD = 0.0056 m, ICC = 0.999; knee angle at third touchdown: RMSD = 1.0°, ICC = 0.994) and therefore confirmed the high reliability of the digitising process.

All further data processing was done in Matlab (v2019a, Natick, MA). de Leva (1996) body segment parameter model was used to obtain data for the whole body CM and for key body segments of interest. A recursive second-order, low-pass Butterworth digital filter (zero phase-lag) was employed to filter the raw coordinate data for the five joint centres digitised continuously throughout the movement. The cut-off frequencies were calculated (mean 13.3 Hz, range 10.0–15.5 Hz) using residual analysis (Winter, 2009).

**Figure 2:**
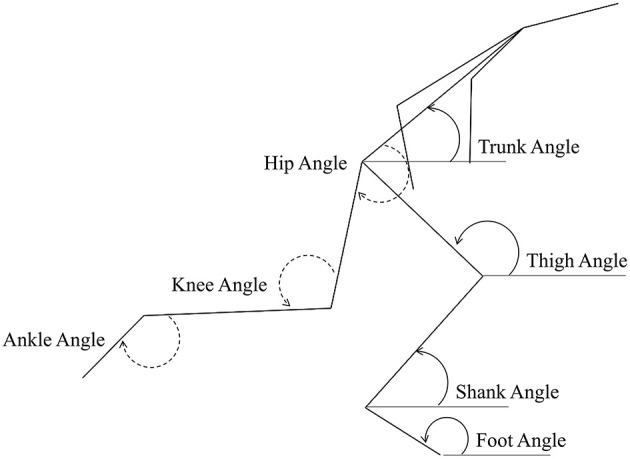
Segment and joint angle definitions used in the study. Segment angles are represented on the ground leg (right, solid line), and joint angles are represented on the swing (left) leg.

For the whole-body analysis at key events, all linear displacement variables (horizontally and vertically for CM and joint centres) were scaled according to the stature of the athletes measured from the digitised data, to account for any differences in height between the two groups. Based on the approach of Ciacci et al. (2017), the sum of the length of shank, thigh and trunk segments was calculated for each athlete for all frames. All linear displacements were divided by this individual scaling factor (mean: 1.359 m for sprinters and 1.456 m for hurdlers), and are therefore presented as dimensionless values. Block spacings were calculated based on the coordinates of the two MTP joint centres in the set position, and were not scaled to stature. Segment angles were defined in an anticlockwise direction relative to the global forward horizontal, and joint angles were defined with extension as positive (see Figure 2).

For the continuous data analysis of the shoulder, hip, knee, ankle and MTP joints on the side of the rear leg in the blocks, all relevant data (joint centre coordinates and segment angles) were time normalised based on key events relevant to the rear foot in the blocks. Those six events were FM, RFBE, GC1_TD_, GC1_TO_, GC3_TD_, and GC3_TO_. Between each successive event the data were time-normalised to 101 data points using a cubic spline. This gave a total of 501 data points from FM to GC3_TO_. The mean value of the time of FFBE for each group was calculated as a percentage between the RFBE and GC1_TD_ events.

All between group comparisons were made using group means and standard deviations (SD), with unpaired samples 95% confidence intervals calculated, based on the differences between the two groups (Altman and Gardner, 2000). All differences were calculated as sprinters minus hurdlers. Group responses were considered different where the 95% confidence intervals did not cross zero (Altman and Gardner, 2000), for both discrete and continuous data. Analysis of discrete data was supplemented with effect size (Cohen's *d*) calculations, with mean and pooled SD calculated according to Altman and Gardner (2000). The effect size magnitude was classified according to the scale proposed by Hopkins et al. (2009). Positive effect sizes represented comparisons where sprinters had a larger value than hurdlers, and negative effect sizes represented comparisons where hurdlers had a larger value than sprinters. Data are presented in the results as (difference in means, 95% confidence interval, effect size classification), according to Altman and Gardner (2000).

#### Intra-Limb Coordination Analysis

**Figure 3:**
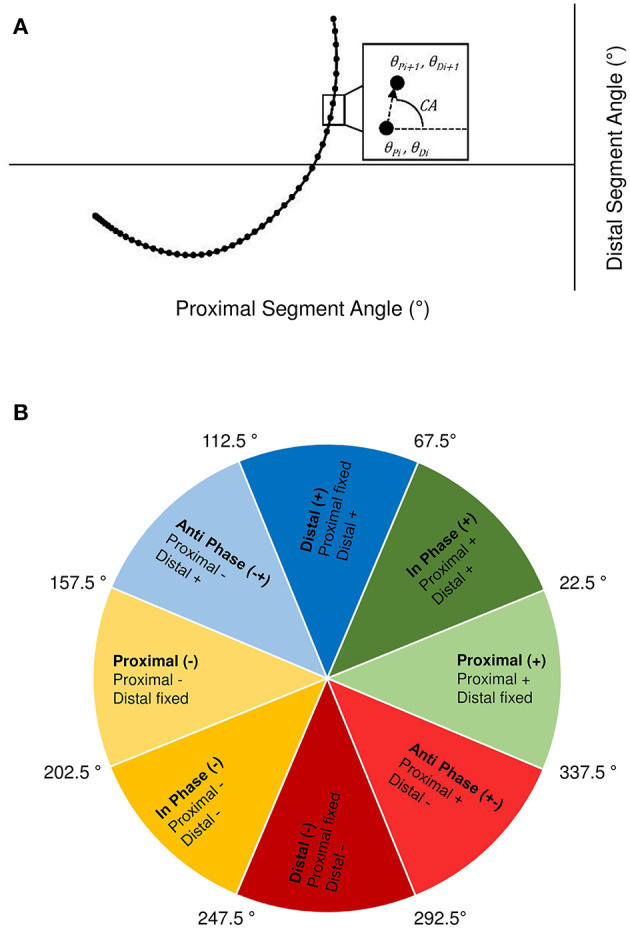
Definition of the coupling angle (CA) from segmental angle-angle plots **(A)** and classification of coordination patterns into distinct “bins” based on the relative motion of the proximal and distal segment **(B)**. Anticlockwise and clockwise segment rotation are defined as positive (+) and negative (–), respectively.

Vector coding techniques (Chang et al., 2008; Needham et al., 2014) were applied to individual and ensemble group mean angle-angle plots for the trunk-thigh, trunk-shank and thigh-shank couples, to obtain the coupling angle (CA) at each instance of the normalised time cycle between FM and GC3_TO_. Specifically, CA was calculated as the orientation of the vector between two adjacent points on the angle-angle plot, relative to the right horizontal and expressed between 0 and 360° (Figure 3A). CA data were then “binned” into one of eight distinct coordination patterns (Chang et al., 2008) based on each segment's relative motion (Figure 3B), with each coordination pattern assigned a specific colour. The colour assignment could then be used to profile coordination across the normalised time cycle to aid visual identification of coordination differences within and between each group. To quantify overall differences in coordination between the sprint and hurdle groups, a coupling angle difference (CA_DIF_) was calculated, by computing a “difference score” in coordination pattern (bin), ranging from 0 (same bin) to 4 (opposite bin) at each instance across the normalised time cycle (Figure 3B). The sum of each difference score was then expressed as a percentage of the maximum possible value, with a lower CA_DIF_ representing closer similarity in coordination patterns.

## Results

### Discrete Analysis at Key Events

**Figure 4:**
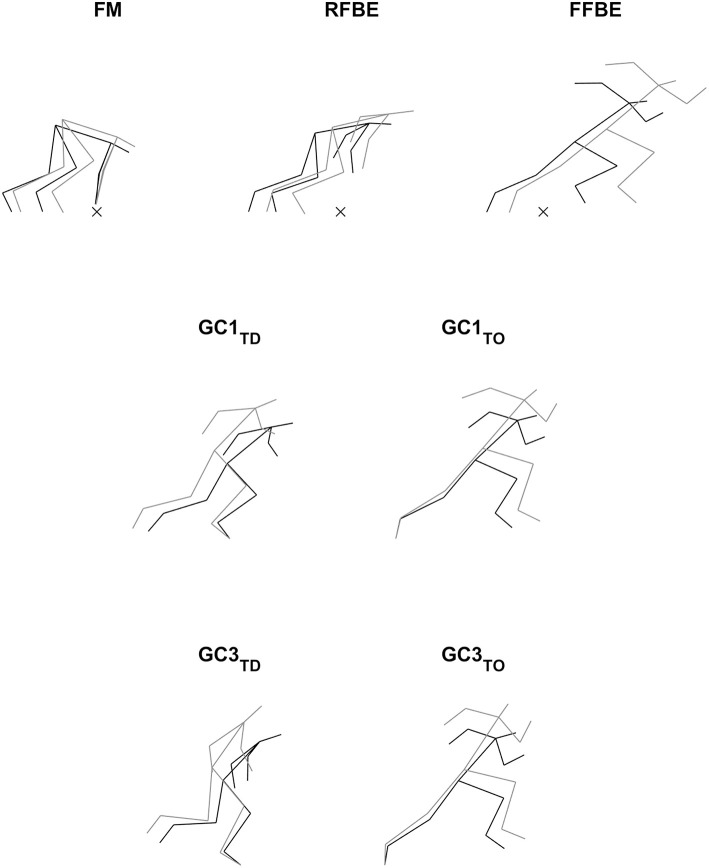
Stick figures showing absolute mean segmental orientations of sprinters (black) and hurdlers (grey) at key events related to the rear leg in the blocks. Block phase events are aligned according to the mean locations of the metacarpal-phalangeal joint centres, with the start line marked (×). Touchdown and take-off events are aligned to the mean locations of the MTP joint of the ground contact leg.

Visual inspection of whole body positions adopted by the two groups at key events (Figure 4) showed differences in block spacings and thigh angles at first movement, but otherwise similar patterns were observed at block exit events. At subsequent touchdown and take-off events sprinters' trunk and shank segments were generally more forward orientated than hurdlers'. Additional temporal analysis (Table 2) showed that although reaction time was identical, hurdlers spent longer in both double- and single-leg push (double-leg difference −0.023 s, −0.043 to −0.003, large effect; single-leg difference −0.031 s, −0.050 to −0.012, large effect) and the total block phase (difference −0.054 s, −0.080 to −0.028, very large effect). With the exception of contact time for ground contacts two and four, all contact and flight times were longer in the hurdlers (medium to very large effects). Overall, the total time to the fourth take-off was greater in the hurdlers than the sprinters (difference −0.228 s, −0.268 to −0.188, extremely large effect). The hurdlers' blocks were set up with both feet nearer to the start line (front foot difference 0.24 m, 0.16 to 0.33, very large effect; rear foot difference 0.13 m, 0.03 to 0.24, large effect), and a greater spacing between the two blocks (difference −0.10 m, −0.17 to −0.04, large effect).

**Table 2:** Durations of key phases from the starting gun to the end of the fourth foot contact, and block spacing distances, for sprinters and hurdlers (Mean ± SD).

**Variable**	**Sprinters**	**Hurdlers**	**Difference**	**95% Confidence interval**	**Effect size (*d*)**
Reaction time [s]	0.155 ± 0.010	0.155 ± 0.018	<0.001	−0.016 to 0.016	0.01^ND^
Double-leg push time [s]	0.194 ± 0.018	0.217 ± 0.018	−0.023	−0.043 to −0.003^*^	−1.29^L^
Single-leg push time [s]	0.148 ± 0.018	0.179 ± 0.016	−0.031	−0.050 to −0.012^*^	−1.81^L^
Total block time [s]	0.497 ± 0.027	0.551 ± 0.018	−0.054	−0.080 to −0.028^*^	−2.32^VL^
Flight time 1 [s]	0.044 ± 0.012	0.077 ± 0.016	−0.033	−0.048 to −0.018^*^	−2.39^VL^
Contact time 1 [s]	0.175 ± 0.014	0.210 ± 0.034	−0.035	−0.064 to −0.007^*^	−1.40^L^
Flight time 2 [s]	0.058 ± 0.012	0.083 ± 0.013	−0.025	−0.040 to −0.011^*^	−1.99^L^
Contact time 2 [s]	0.171 ± 0.014	0.165 ± 0.016	0.006	−0.011 to 0.023	0.41^S^
Flight time 3 [s]	0.063 ± 0.012	0.099 ± 0.012	−0.037	−0.050 to −0.023^*^	−2.96^VL^
Contact time 3 [s]	0.138 ± 0.011	0.154 ± 0.018	−0.017	−0.033 to −0.001^*^	−1.16^M^
Flight time 4 [s]	0.072 ± 0.010	0.099 ± 0.010	−0.027	−0.038 to −0.016^*^	−2.78^VL^
Contact time 4 [s]	0.136 ± 0.016	0.142 ± 0.008	−0.006	−0.020 to 0.008	−0.48^S^
Total time to end of fourth contact [s]	1.352 ± 0.040	1.580 ± 0.029	−0.228	−0.268 to −0.188^*^	−6.40^EL^
Front foot toe distance from start line in blocks [m]	0.56 ± 0.05	0.33 ± 0.08	0.24	0.16 to 0.33^*^	3.36^VL^
Back foot toe distance from start line in blocks [m]	0.84 ± 0.05	0.71 ± 0.12	0.13	0.03 to 0.24^*^	1.40^L^
Block spacing [m]	0.28 ± 0.03	0.38 ± 0.08	−0.10	−0.17 to −0.04^*^	−1.70^L^

*Differences are calculated as sprinters minus hurdlers, so negative values represent values for hurdlers being greater than sprinters*.

* *Represents where the confidence interval for the between group comparison does not cross zero (Altman and Gardner, 2000). Effect size scale (Hopkins et al., 2009): No Difference (ND) = < 0.20; Small (S) = ≥ 0.20 to < 0.60; Moderate (M) = ≥ 0.60 < 1.20; Large (L) = ≥ 1.20 to < 2.00; Very Large (VL) = ≥ 2.00 to < 4.00; Extremely Large (EL) = ≥ 4.00*.

**Figure 5:**
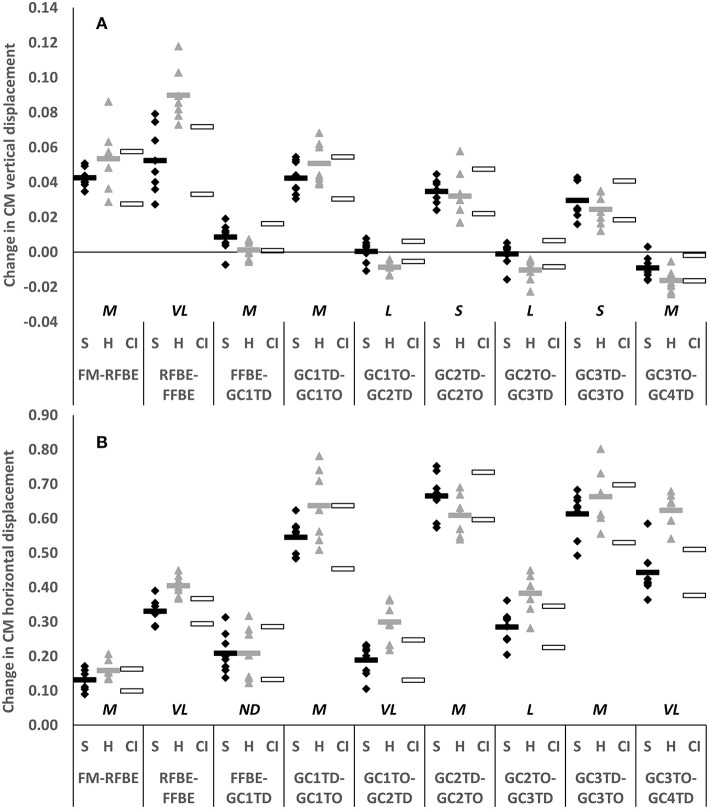
Change in vertical **(A)** and horizontal displacement **(B)** of whole body CM of sprinters (black diamonds) and hurdlers (grey triangles) between successive key events, scaled to stature. Group means are represented by shaded rectangles, and 95% confidence intervals with white rectangles. Values for hurdlers are different from sprinters where the hurdlers mean falls outside the 95% confidence intervals [i.e., where the confidence interval for the between group comparison does not cross zero (Altman and Gardner, 2000)]. Effect size classifications are represented above x-axis labels (Hopkins et al., 2009): No Difference (*ND*) = < 0.20; Small (*S*) = ≥ 0.20 to < 0.60; Moderate (*M*) = ≥ 0.60 < 1.20; Large (*L*) = ≥ 1.20 to < 2.00, Very Large (*VL*) = ≥ 2.00 to < 4.00; Extremely Large (*EL*) = ≥ 4.00.

**Figure 6:**
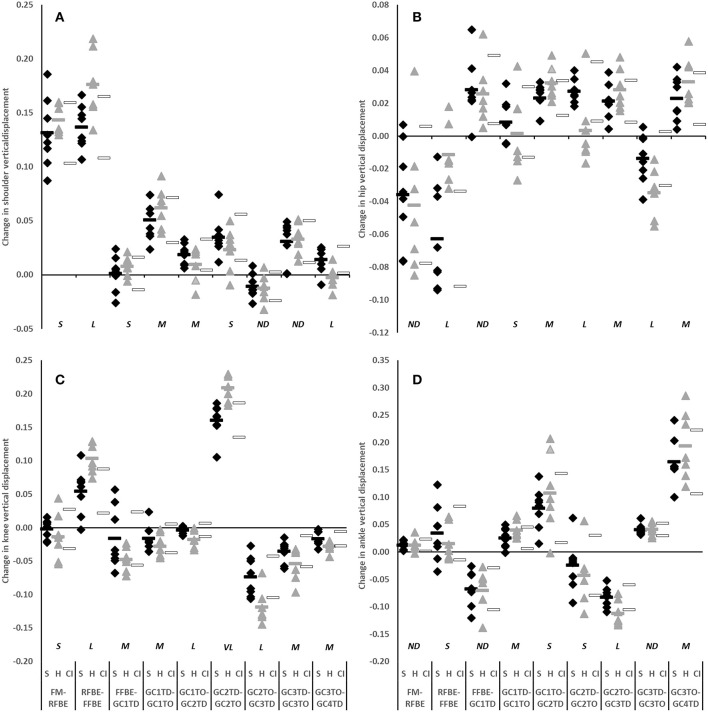
Change in vertical displacement of shoulder **(A)**, hip **(B)**, knee **(C)**, and ankle **(D)** of sprinters (black diamonds), and hurdlers (grey triangles) between successive key events, scaled to stature. Group means are represented by shaded rectangles, and 95% confidence intervals with white rectangles. Values for hurdlers are different from sprinters where the hurdlers mean falls outside the 95% confidence intervals [i.e., where the confidence interval for the between group comparison does not cross zero (Altman and Gardner, 2000)]. Effect size classifications are represented above x-axis labels (Hopkins et al., 2009): No Difference (*ND*) = < 0.20; Small (*S*) = ≥ 0.20 to < 0.60; Moderate (*M*) = ≥ 0.60 < 1.20; Large (*L*) = ≥ 1.20 to < 2.00, Very Large (*VL*) = ≥ 2.00 to < 4.00; Extremely Large (*EL*) = ≥ 4.00.

The clearest differences in translation of the CM occurred during the block phase, particularly after rear foot block exit, and during the flight phases succeeding each post-block foot contact. Hurdlers displaced their CM vertically and horizontally more than sprinters between RFBE and FFBE (vertical difference −0.037, −0.057 to −0.018, very large effect; horizontal difference −0.075, −0.111 to −0.038, very large effect, Figures 5A,B). Differences in CM vertical displacement changes between RFBE and FFBE were mirrored in changes at the shoulder (vertical difference −0.039, −0.068 to −0.011, large effect), hip (vertical difference −0.051, −0.080 to −0.022, large effect) and knee (vertical difference −0.049, −0.082 to −0.016, large effect) during the same phase, which were all greater in hurdlers than sprinters (Figures 6A–C).

Whilst there was a clear overlap in confidence intervals for change of CM vertical displacement between FM and RFBE (difference −0.011, −0.026 to 0.004, moderate effect, Figure 5A), the range of responses was clearly greater in hurdlers than sprinters, indicating more variability within the hurdler group (SD hurdlers 0.019, SD sprinters 0.005). This was reflected in more variability in the hurdlers in the vertical displacement of both hip (SD hurdlers 0.043, SD sprinters 0.031) and knee joint centres (SD hurdlers 0.035, SD sprinters 0.014), but less variability in the raising of the shoulder (SD hurdlers 0.014, SD sprinters 0.032). At each of those joints there was a clear overlap in the confidence intervals for the magnitude of the change in CM vertical displacement (small effect, shoulder and knee; no difference, hip; Figures 6A–C).

**Figure 7:**
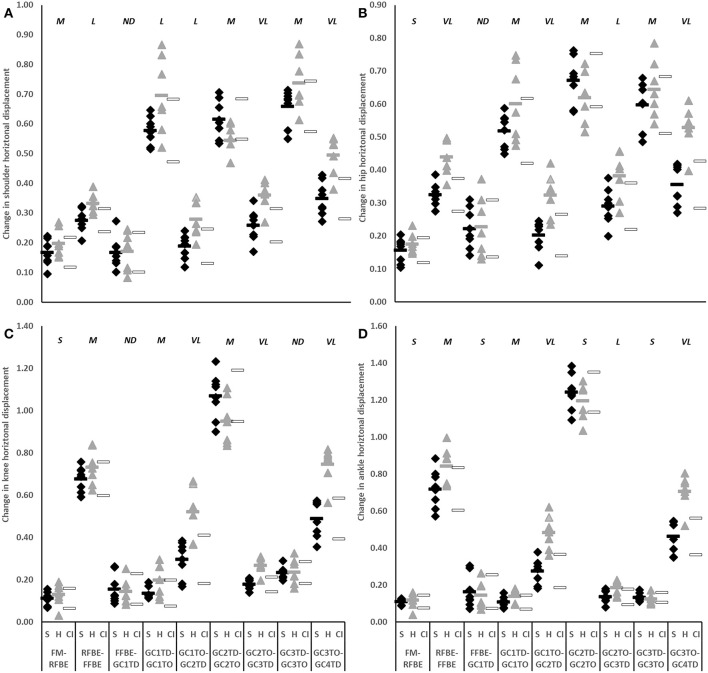
Change in horizontal displacement of shoulder **(A)**, hip **(B)**, knee **(C)**, and ankle **(D)** of sprinters (black diamonds), and hurdlers (grey triangles) between successive key events, scaled to stature. Group means are represented by shaded rectangles, and 95% confidence intervals with white rectangles. Values for hurdlers are different from sprinters where the hurdlers mean falls outside the 95% confidence intervals [i.e., where the confidence interval for the between group comparison does not cross zero (Altman and Gardner, 2000)]. Effect size classifications are represented above x-axis labels (Hopkins et al., 2009): No Difference (*ND*) = < 0.20; Small (*S*) = ≥ 0.20 to < 0.60; Moderate (*M*) = ≥ 0.60 < 1.20; Large (*L*) = ≥ 1.20 to < 2.00, Very Large (*VL*) = ≥ 2.00 to < 4.00; Extremely Large (*EL*) = ≥ 4.00.

During initial acceleration hurdlers produced greater horizontal CM displacement than sprinters in each of the second to fourth flight phases (GC1_TO_-GC2_TD_ difference −0.110, −0.169 to −0.052, very large effect; GC2_TO_-GC3_TD_ difference −0.097, −0.157 to −0.037, large effect; GC3_TO_-GC4_TD_ difference −0.181, −0.247 to −0.114, very large effect; Figure 5B). These group-level differences in change in CM horizontal displacement were reflected by the shoulder, hip, knee, and ankle in each of the second, third and fourth flight phases (large to very large effects, Figure 7).

### Continuous and Angular Analysis

**Figure 8:**
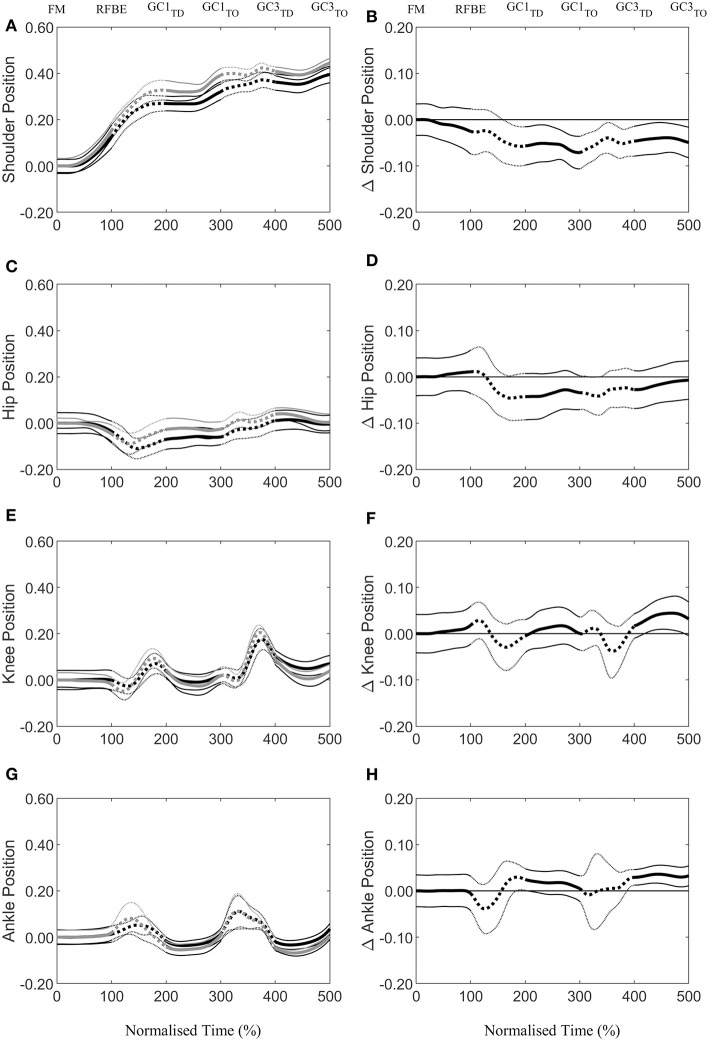
Continuous vertical position relative to the set position (Mean ± SD) of the shoulder **(A)**, hip **(C)**, knee **(E)**, and ankle **(G)**, scaled to individual stature for sprinters (black) and hurdlers (grey), and corresponding differences **(B,D,F,H)** between the sprinters, and hurdlers (thick line), and 95% confidence interval (thin line). Positive differences indicate a greater value for sprinters, and negative differences a greater value for hurdlers. Sections where the confidence interval bands do not cross zero (x-axis) represent clear differences between groups. Solid lines represent the double-leg push phase in the blocks, and first and third ground contacts, whereas dashed lines represent the first and second swing phases of the rear leg, with key events related to the rear leg in the blocks marked above the figure.

**Figure 9:**
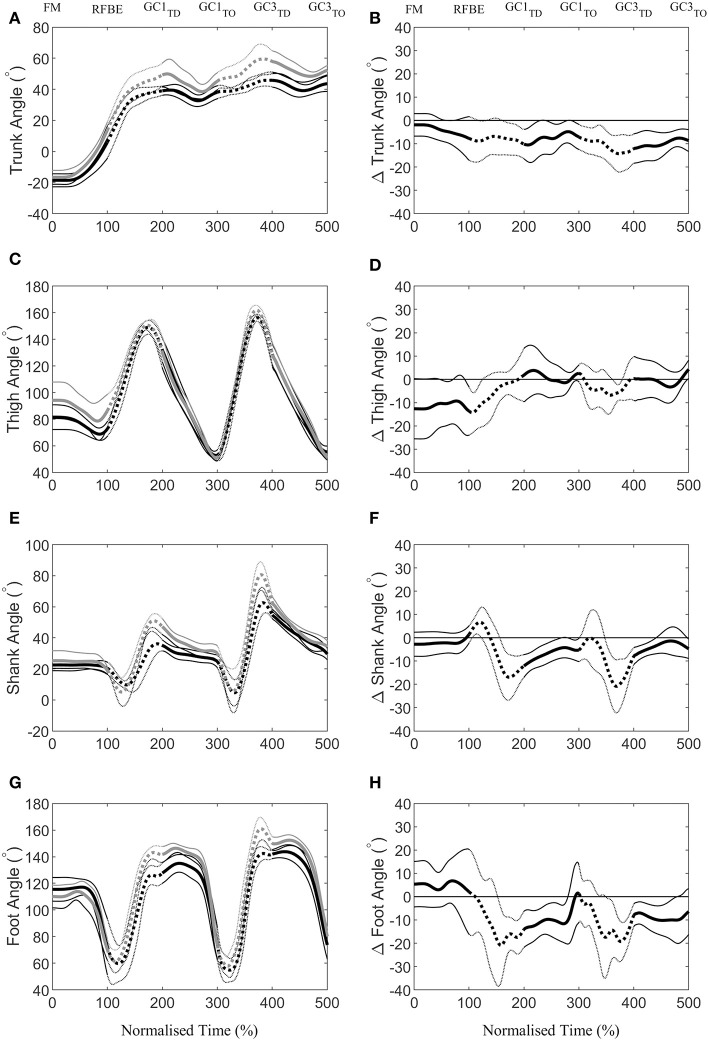
Continuous segment angles (Mean ± SD) of the trunk **(A)**, thigh **(C)**, shank **(E)**, and foot **(G)**, for sprinters (black) and hurdlers (grey), and corresponding differences **(B,D,F,H)** between the sprinters, and hurdlers (thick line), and 95% confidence interval (thin line). Positive differences indicate a greater value for sprinters, and negative differences a greater value for hurdlers. Sections where the confidence interval bands do not cross zero (x-axis) represent clear differences between groups. Solid lines represent the double-leg push phase in the blocks, and first and, third ground contacts, whereas dashed lines represent the first and second swing phases of the rear leg, with key events related to the rear leg in the blocks marked above the figure. For definitions of segment angles (see Figure 2).

During the first swing phase (at 160% normalised time from FM to GC3_TO_ i.e., at 60% time from RFBE to GC1_TD_), the hurdlers had raised their shoulders to a greater extent, when accounting for stature, and maintained this through the remainder of the initial acceleration phase (Figure 8B). There were no clear differences in joint and segment angles of the rear leg in the set position, but moderate effect sizes for a more flexed hip and vertical thigh in the hurdlers (Figure 9, Table 3). The front leg hip angle was more flexed in the hurdlers in the set position (difference 10°, 2 to 18, large effect), with no clear differences but a moderate effect size for hurdlers' thigh being more horizontal and shank being more vertical (Figure 9, Table 3).

**Table 3:** Joint and segment angles in the set position, and range of motion throughout initial acceleration for sprinters and hurdlers (Mean ± SD).

**Variable**	**Sprinters**	**Hurdlers**	**Difference**	**95% Confidence interval**	**Effect size (*d*)**
**Set position angle (°)**					
Rear hip	80 ± 9	69 ± 11	11	0 to 22	−1.10^M^
Rear knee	121 ± 10	111 ± 19	10	−7 to 27	−0.66^M^
Rear ankle	87 ± 9	95 ± 11	−8	−20 to 3	−0.80^M^
Rear thigh	82 ± 9	94 ± 14	−13	−26 to 0	−1.10^M^
Rear shank	22 ± 2	25 ± 6	−3	−8 to 2	−0.60^M^
Rear foot	116 ± 9	110 ± 9	−5	−4 to 15	0.61^M^
Front hip	45 ± 7	35 ± 8	10	2 to 18^*^	1.41^L^
Front knee	93 ± 5	90 ± 10	3	−6 to 11	0.35^S^
Front ankle	102 ± 8	98 ± 11	4	−6 to 15	0.46^S^
Front thigh	118 ± 6	126 ± 12	−8	−19 to 2	−0.89^M^
Front shank	31 ± 5	36 ± 5	−6	−11 to 0	1.13^M^
Front foot	108 ± 7	118 ± 8	−10	−18 to −2^*^	1.32^L^
Trunk	−19 ± 4	−17 ± 4	−2	−7 to 3	−0.44^S^
**Range of motion (°)**					
***FM - RFBE***					
Hip	33 ± 18	38 ± 9	−5	−21 to 11	−0.33^S^
Knee	7 ± 11	2 ± 17	5	−11 to 21	0.36^S^
Ankle	41 ± 14	34 ± 13	7	−8 to 23	0.55^S^
Trunk	25 ± 10	30 ± 7	−6	−15 to 4	−0.68^M^
Thigh	−9 ± 10	−8 ± 13	−1	−14 to 12	−0.09^ND^
Shank	−2 ± 2	−6 ± 5	4	0 to 9	1.03^M^
Foot	−43 ± 16	−40 ± 12	−3	−19 to 13	−0.23^S^
***FM-GC1**_***TD***_*					
Trunk	59 ± 6	67 ± 10	−8	−17 to 1	0.99^M^
***GC1**_***TD***_**- GC1**_***TO***_*					
Hip	78 ± 11	76 ± 8	2	−9 to 13	0.24^S^
Knee	69 ± 13	62 ± 4	6	−5 to 17	0.62^M^
Ankle	43 ± 11	51 ± 6	−8	−19 to 2	−0.92^M^
Trunk	−1 ± 3	−4 ± 4	3	−1 to 7	0.91^M^
Thigh	−79 ± 11	−80 ± 11	1	−11 to 13	0.07^ND^
Shank	−10 ± 5	−17 ± 8	7	0 to 14	1.06^M^
Foot	−53 ± 14	−68 ± 11	15	1 to 30^*^	1.19^M^
***GC3**_***TD***_**- GC3**_***TO***_*					
Hip	71 ± 11	72 ± 9	0	−12 to 11	−0.05^ND^
Knee	48 ± 8	49 ± 4	−1	−8 to 7	−0.11^ND^
Ankle	43 ± 8	41 ± 5	2	−6 to 9	0.23^S^
Trunk	−2 ± 2	−6 ± 7	3	−2 to 9	0.74^M^
Thigh	−73 ± 10	−77 ± 9	4	−7 to 15	0.42^S^
Shank	−25 ± 3	−29 ± 7	3	−3 to 9	0.62^M^
Foot	−68 ± 8	−70 ± 9	2	−8 to 11	0.20^S^

*Differences are calculated as sprinters minus hurdlers, so negative values represent values for hurdlers being greater than sprinters*.

* *Represents where the confidence interval for the between group comparison does not cross zero (Altman and Gardner, 2000). Effect size scale (Hopkins et al., 2009): No Difference (ND) = < 0.20; Small (S) = ≥ 0.20 to < 0.60; Moderate (M) = ≥ 0.60 < 1.20; Large (L) = ≥ 1.20 to < 2.00; Very Large (VL) = ≥ 2.00 to < 4.00; Extremely Large (EL) = ≥ 4.00*.

After FM, hurdlers briefly had a more vertically orientated thigh segment than sprinters during the double-leg push phase (from 47 to 71% time) and around the transition from double- to single-leg push (93–124% time, Figure 9D). During the initial acceleration phase, hurdlers had more vertically orientated trunk and shank segments, as well as a more horizontally orientated foot segment late in the first swing and early into the first ground contact phase (trunk, 161–230% and 239–279%, Figure 9B; shank 156–268%, Figure 9F; foot 153–280%, Figure 9H). These differences at the trunk, shank, and foot occurred again late in the second swing phase and into the third ground contact (trunk, 286–500%, Figure 9B; shank 348–440%, Figure 9F; foot 349–479%, Figure 9H), and continued thereafter at the trunk, with a more upright posture maintained throughout the initial acceleration phase. The thigh segment was briefly more upright in the hurdlers late in the second swing phase (from 361 to 379% time, Figure 9D). The only clear difference in the ranges of motion of joints or segments between each of the key events investigated, was at the foot during the first ground contact (difference 15°, 1 to 30, moderate effect, Table 3). There were, however, also moderate effect sizes for range of motion of the trunk segment between FM and both RFBE and GC1_TD_, with hurdlers showing greater values (FM-RFBE difference −6°, −15 to 4, moderate effect; FM-GC1_TD_ difference −8°, −17 to 1, moderate effect).

### Intra-Limb Segmental Coordination

#### Trunk-Thigh

**Figure 10:**
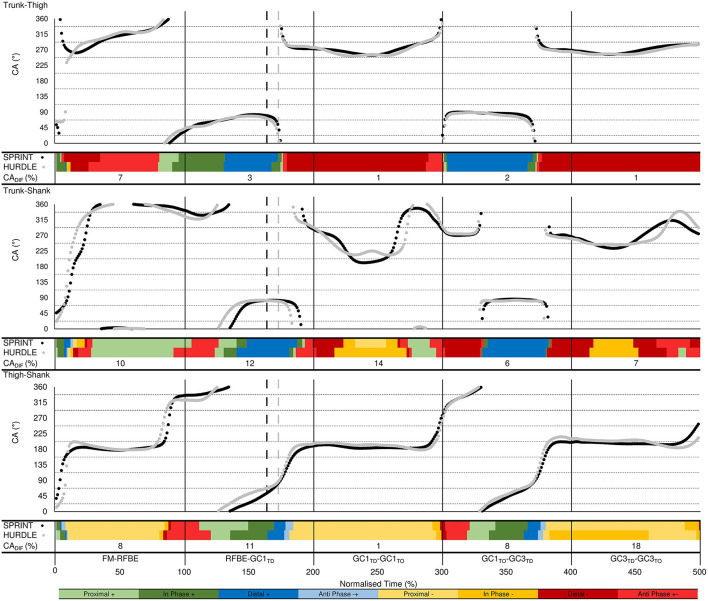
Trunk-thigh (top), trunk-shank (middle), and thigh-shank (bottom) coupling angle-normalised time profiles for the sprint (black dots) and hurdle (grey dots) groups throughout each key phase of start and initial acceleration related to the rear leg in the blocks. Colour profiles represent coupling angle classification at each instance across the normalised time cycle (see Figure 3B). The overall difference score (CA_DIF_) in coordination patterns between the sprint and hurdle groups is shown beneath each colour profile. Group mean instances of FFBE are indicated by black (sprint) and grey (hurdle) vertical dashed lines.

**Figure 11:**
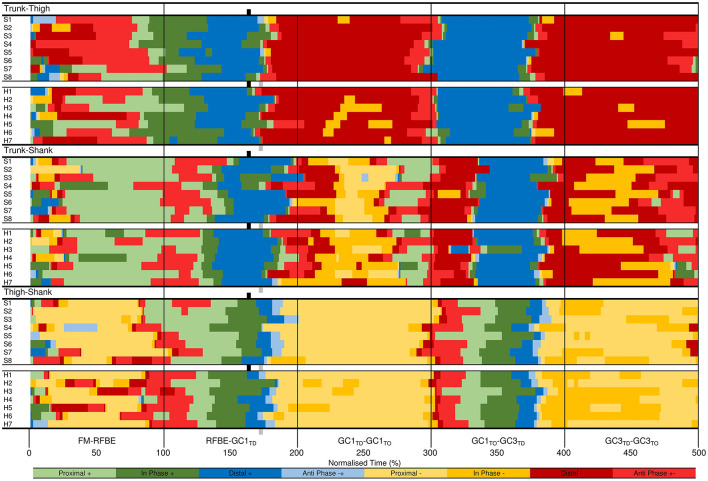
Individual coordination profiles for the trunk-thigh (top), trunk-shank (middle), and thigh-shank (bottom) couples, throughout each key phase of start and initial acceleration related to the rear leg in the blocks. Colour profiles represent coupling angle classification at each instance across the normalised time cycle (see Figure 3B). Group mean instances of FFBE are indicated by black (sprint) and grey (hurdle) shading surrounding each group of individual athletes.

Coordination of the trunk-thigh segments was similar between groups, with CA_DIF_ magnitudes of 7% during the double-leg push phase and 1–3% after RFBE (Figure 10). The greater difference during the double-leg push was mainly attributed to the onset of movement. From FM, sprinters exhibited an earlier transition and longer duration of distal (–) coordination (dark red), whereas hurdlers spent a greater duration of this initial movement in coordination patterns dominated by anticlockwise trunk rotation (anti phase +−, light red; in-phase +, dark green, Figure 10). During GC_1_ and GC_3_ both groups exhibited a dominance of distal (–) coordination as the thigh rotated clockwise and trunk angle remained relatively fixed (Figures 10, 11).

#### Trunk-Shank

The greatest inter-group and inter-individual differences in trunk-shank coordination were again attributed to the onset of movement (Figures 10, 11). Of all phases during initial acceleration CA_DIF_ was higher during the double-leg push phase (10%), first swing (12%) and GC_1_ (14%), although coordination patterns between the two groups were often within one bin (Figure 10). An earlier onset of anticlockwise shank rotation after RFBE in the hurdle group resulted in an earlier transition away from anti phase (+−) coordination, through to in-phase (+) and eventually distal (+) coordination once the trunk ceased anticlockwise rotation (Figure 10). During GC_1_, group differences in coordination arose around mid-stance, with sprinters and hurdlers showing proximal (–) and in phase (–) patterns, respectively. Individual analysis (Figure 11) again highlighted the overall consistency within and between groups with all athletes adopting the same main patterns of coordination after movement onset. Individual differences manifested themselves through temporal shifts between coordination patterns and the time spent in each, as opposed to any clear differences in the major coordination patterns adopted.

#### Thigh-Shank

At a group level, the largest CA_DIF_ was apparent during GC_3_ (18%) as patterns of proximal (–) and anti phase (–+) dominated for the sprint and hurdle groups, respectively, although absolute differences in the coupling angle were small (Figure 10). Once more, both group and individual analyses highlighted the overall consistency in coordination patterns, with temporal differences in the transition between major coordination patterns dictating inter-group and inter-individual differences (Figures 10, 11).

## Discussion

The first aim of this study was to quantify and explain the start and initial acceleration technique of the world's best male sprinters and hurdlers *in situ* in an elite competition environment. This is the first time in the biomechanics research literature that such data have been captured live, in this case from the finals of the 2018 IAAF World Indoor Championships, enabling a novel approach to examining an important aspect of sprint acceleration performance. Secondly, based on a comparison of the sprinters and hurdlers, the aim was to elucidate the mechanisms by which the athletes translate their CM during the start and initial acceleration phases. Despite the different task constraints placed on the athletes by the two events, there were many similarities between the kinematic and inter-segment coordination profiles of the world-class sprinters and hurdlers investigated in the current study.

Overall, the similarity in coordination patterns found in this study (Figures 10, 11) is an important and novel finding that will be particularly useful for scientists, coaches and athletes. The world-class sprinters and hurdlers studied here organised their lower limb and trunk movement in a similar manner, and this contributes new conceptual understanding of the mechanisms that underpin start and initial acceleration performance. The differences in the raising of the CM are unlikely to be a result of differences in coordination through the start and initial acceleration phase, but result from small differences in the set position and a summation of small to moderate differences in extension range of motion throughout each push-off phase (Table 2). Those differences in the set position often come about as a strategic decision by coach and hurdler, to ensure that the athlete has less total distance to cover to reach the first hurdle (Mann and Murphy, 2018).

Following a near-identical reaction time in the sprinters and hurdlers, phase times were longer in hurdlers than sprinters in all phases up to take-off from the fourth contact, with the exception of the contact times when the front leg in the blocks was in contact with the track. These longer phase durations (Table 2), in addition to the hurdlers displacing their CM more horizontally during the single-leg push on the blocks and flight phases following the first post-block ground contact (Figure 5), might be a consequence of all hurdlers adopting a seven-step approach to the first hurdle. However, with no hurdlers electing to adopt an eight-step approach in this final, a direct comparison of the two strategies is not possible. Nonetheless, the seven-step approach to the first hurdle demands steps in the initial acceleration phase on average to be lengthened, both spatially and temporally (Mann and Murphy, 2018), and that was clearly evident here in comparison to the sprinters. It was interesting to note that the contact times that showed no difference between hurdlers and sprinters were those that were taken with what would be the lead leg during subsequent hurdle clearances. It is beyond the scope of this study to investigate the differing roles of lead and trail legs further, but it is an interesting avenue for future research, especially given the asymmetrical and repetitive nature of the hurdle-unit gait cycle.

The hurdlers used a larger block spacing than the sprinters (difference −0.10 m, −0.17 to −0.04, large effect, Table 1). According to block spacing classifications typically used, the hurdlers' mean spacing was medium, whilst the sprinters' was bunched (Slawinski et al., 2012). Despite the effects of relative block spacing being well-known (e.g., Henry, 1952; Slawinski et al., 2012), and supported in this study with greater spacings leading to longer push-phase times, the absolute positioning of the blocks from the start line has received little attention in the biomechanics research literature (Schot and Knutzen, 1992). Coaching literature has identified that hurdlers tend to place starting blocks closer to the start line than sprinters, but that this has negative consequences for performance (Mann and Murphy, 2018). In this study, the hurdlers' medium block spacing combined with the front block being closer to the start line (Table 1) led to relatively more flexed hip angles in the set position (front hip difference 10°, 2 to 18, large effect; rear hip difference 11°, 0 to 22, medium effect; Figure 4, Table 3). The net effect would have led to a greater extensor angular velocity of the front hip in the hurdlers, particularly during the single-leg push phase (Slawinski et al., 2013), which therefore would have increased the vertical displacement of the CM and shoulder joint centres (Figures 6A, 7A, respectively) more so in the hurdlers than sprinters. Analysis of trunk-thigh couples in the block phase showed an earlier onset of anti phase (+−) coordination (Figure 10), supporting the finding that hurdlers started to raise their trunk and CM earlier in the block phase than sprinters. Indeed, the biggest differences seen in the raising of the CM between hurdlers and sprinters occurred during the block phase, and likely come as a direct consequence of the differences in body orientations in the set position.

During the double-leg push phase the hurdlers as a group were clearly more variable than the sprinters in the amount that they raised the CM, and hip and knee joint centres, but clearly less variable in the amount that they raised their shoulder joint centre (Figures 6, 7). This suggests a more variable response in the lower limb within the hurdlers, between different athletes, and highlights individual variations in the responses to the task. It is important for coaches to note that despite the consistent extent to which the hurdlers raised their shoulders during the double leg push phase, the manner in which this was controlled by the distal segments was much more variable.

Continuous segmental data (Figures 4, 8, 9) revealed that the hurdlers had more vertically orientated trunk and shank segments, and more horizontally orientated foot segments, than sprinters for periods in late swing and early stance around both GC1_TD_ and GC3_TD_. At each of the subsequent take-off events the differences in trunk, shank and foot angles between hurdlers and sprinters, whilst still clear, had reduced in magnitude (Figure 9). It therefore appears that these differences tend to accumulate during the longer flight phases in the hurdlers (Table 1), but then during the ground contact phases the hurdlers bring their segment orientations back towards those adopted by the sprinters (Figure 9). It is likely that the differences in segment angles combined with the greater stature of the hurdlers combine to provide the visual impression of a more upright stance in the hurdlers, particularly at touchdown events.

One key novel aspect of the current study was the complimentary nature of both qualitative and quantitative analysis of coordination, utilising popular ‘binning' approaches (Silvernail et al., 2018) to visualise local and global similarities and differences in inter-segmental coordination in this unique sample of world-class athletes. The largest inter-group and inter-individual differences in coordination were observed soon after FM. As coordination variability has been shown to increase during changes to the state of the system (Heiderscheit et al., 2002), it could be theorised that the abrupt state change at FM contributed to the observed inter-group and inter-individual differences. In addition, the differences in the set position between athletes might have dictated initial coordination of the system, as shown by Gheller et al. (2015) who found that starting knee angle influenced coordination patterns during vertical jumps. The reduction in inter-individual differences after the initial part of the double-leg push phase could be indicative of self-organisation towards task-specific coordinative structures (Newell, 1986). However, it should be recognised that artefacts when consecutive data points are in close proximity (Heiderscheit et al., 2002) could also have influenced the initial inter-group and inter-individual differences in coordination at this early stage of the movement.

The nature of the data presented in this study, captured *in situ* in the world's best sprinters and hurdlers during the finals of the IAAF World Indoor Championships ensured that the ecological validity of the analysis conducted here was maintained in an exceptional manner. Indeed, the 60 m final included three of the fastest twenty times run in the history of the event. The very nature of this maintenance of ecological validity means that the sample sizes were small. However, the populations of the very best athletes in the world in any individual event are by definition small, and this study provides a comprehensive analysis of the mechanisms for the translation of the CM in this world-class sample. In doing so, it therefore addresses a significant gap in the biomechanics research literature. Further, studies of sprint hurdle biomechanics have generally focused on the hurdle clearance and three-step inter-hurdle cycle (e.g., Mann and Herman, 1985a; McDonald and Dapena, 1991; Salo et al., 1997). To the authors' knowledge this is the first study that has investigated the start and initial acceleration phase in elite high hurdlers, as well as being the first to apply coordination analyses to the start and initial acceleration phase of a sprint. Data presented here have shown that the biggest differences in the raising of the CM occurred during the single-leg push phase in the blocks. Future research should therefore additionally consider the role of the front leg in the blocks, that might reveal interesting additional insights into the mechanisms utilised by sprinters and hurdlers.

### Coaching Implications

The similarities that were shown between the sprinters and hurdlers, despite the differing task constraints being faced by the athletes, were the most striking feature of this analysis. Overall, the response to the task to accelerate the CM in a horizontal direction was primarily the same across the two events, which challenges current thinking. The key differences that did occur came primarily from the initial body positions in the set position, so there should be some consideration for an individual approach to block set up by coaches and athletes. Once the push phase in the blocks began, there was clearly a relatively common pattern of coordination across all athletes. This overall consistent organisation of movement during the start and initial acceleration phase is an important consideration for coaches, and needs to be maintained even when accounting for individual factors that may influence performance such as stature or strength. The hurdlers as a group were more variable than sprinters in the manner in which they raised their lower body segments in the block phase, but then also used the following ground contact phases to converge their segment orientations back towards those adopted by the sprinters. It may therefore be that small visual differences in orientations that might be apparent at touchdown events should not be of major concern, and are typically overcome by hurdlers during the subsequent ground contact phase.

The fact that all seven hurdlers studied here chose to adopt a seven-step approach to the first hurdle meant that a direct comparison of the differences between seven- and eight-step approaches was not possible. Additional work is no doubt required to address the implications of any differences between these two approach strategies. Nonetheless, the data presented here provides an important underpinning to the coaching literature, and reveals that even when high-hurdlers adopt a seven-step approach to the first hurdle, there are many similarities between the techniques they adopt and those of their sprint counterparts.

## Conclusion

This novel study was successful firstly in quantifying and explaining the start and initial acceleration technique of the world's best male sprinters and hurdlers *in situ* in an elite competition environment, and secondly in elucidating the similarities in the mechanisms by which sprinters and hurdlers translate their CM during the start and initial acceleration phases. Coordination patterns adopted by sprinters and hurdlers were similar throughout the start and initial acceleration phases, and differences in CM raising generally occurred as a result of small differences that were present from block set-up. This study has generated an exceptional and significant data set, and the analysis presented here will become a primary source of reference for those wanting to further explore the start and initial acceleration phase in both sprinters and hurdlers as a means of optimising performance. The findings from this study contribute new conceptual understanding of the mechanisms that underpin start and initial acceleration performance for scientists, coaches and athletes.

## Data Availability

The datasets generated for this study will not be made publicly available in order to avoid identifying individual athletes.

## Ethics Statement

The studies involving human participants were reviewed and approved by (1) IAAF obtained signed Athlete Acknowledgment and Agreement Forms from the athletes to use their moving images. (2) The study was approved by the Leeds Beckett University Ethics sub-committee (School local approval by Research Ethics Coordinator). The patients/participants provided their written informed consent to participate in this study.

## Author Contributions

IB, ABr, HL, GP, SM, P-JV, JW, and ABi conceived and designed the study. GP, BH, CT, LP, JW, and ABi performed data collection. IB, ABr, HL, BH, CT, LP, JW, and ABi processed data. IB, ABr, HL, MW, GP, JW, and ABi interpreted the results of the research. IB and ABr drafted the manuscript. IB, ABr, and HL prepared tables and figures. IB, ABr, HL, MW, GP, BH, CT, LP, SM, P-JV, JW, and ABi edited, critically revised, and approved the final version for submission.

### Conflict of Interest Statement

ABi is the Director of Athletics Biomechanics. The remaining authors declare that the research was conducted in the absence of any commercial or financial relationships that could be construed as a potential conflict of interest.
